# Reported Male Circumcision Practices in a Muslim-Majority Setting

**DOI:** 10.1155/2017/4957348

**Published:** 2017-01-17

**Authors:** Abdul Wahid Anwer, Lubna Samad, Sundus Iftikhar, Naila Baig-Ansari

**Affiliations:** ^1^Department of General Surgery, The Indus Hospital, Korangi Crossing, Karachi 75190, Pakistan; ^2^Department of Surgical Oncology, Shaukat Khanum Memorial Cancer Hospital & Research Centre, 7-A Block R-3, Johar Town, Lahore, Pakistan; ^3^Department of Pediatric Surgery, The Indus Hospital, Korangi Crossing, Karachi 75190, Pakistan; ^4^Indus Hospital Research Center, The Indus Hospital, Korangi Crossing, Karachi 75190, Pakistan

## Abstract

*Introduction*. Male circumcision is a recommended practice in Muslim tradition. It is important to ensure that this procedure is performed as safely as possible in these communities.* Methods*. Five hundred adult men and women with at least one male child less than 18 years were interviewed in Karachi, Pakistan, regarding details of their child's circumcision. The survey focused on actual and perceived delays in circumcision and perceptions about appropriate age and reasons and benefits and complications of the procedure. Circumcisions done after two months of age were defined as delayed.* Results*. Religious requirement was the primary reason for circumcision in 92.6% of children. However, 89.6% of respondents were of the opinion that circumcision had medical benefits as well. Half of the children (54.1%) had delayed circumcision (range 2.5 months to 13 years), even though 81.2% of parents were of the opinion that circumcisions should be done within 60 days of birth. Facility-delivered babies had less delay in circumcisions (49.1%) as compared to home-delivered babies (60.5%).* Conclusion*. Understanding the perceptions and practices around male circumcision can help guide national strategies for designing and implementing safe circumcision programs in Muslim-majority settings, with the potential to benefit an annual birth cohort of 20–25 million boys worldwide.

## 1. Introduction

Circumcision is an ancient rite [1, 2] with evidence to suggest that it was practiced in diverse communities including the ancient Egyptians, Australian aborigines, and African bushmen. Today, although male circumcision is one of the most common surgical procedures performed worldwide with an estimated one in three males circumcised globally [3], it continues to be a contentious procedure, with equally strong proponents and opponents of this practice. Over the years, recommendations have changed to reflect the available body of scientific evidence. In its most recently published technical report, the American Academy of Paediatricians summarizes that the health benefits of newborn male circumcision outweigh the risks, thus justifying access to circumcision for consenting families. The specific benefits include reduced risk of acquiring of urinary tract infections, Human Immunodeficiency Virus (HIV) and some other sexually transmitted infections, and penile cancer [4]. In particular, evidence linking male circumcision with decreased transmission of HIV has led the global community to recommend mass circumcision programs as a strategy for HIV prevention [5–7]. The reliability of this data is disputed by some who term this as a violation of a child's rights [3].

While the value of circumcision may be debated in popular and scientific literature, it is a recommended practice in both Jewish as well as Muslim traditions. It is estimated that the prevalence is greater than 98% in Jews living in the UK, United States, and Israel, with an almost universal coverage in Muslim-majority countries and communities with almost two-thirds of circumcised males worldwide being Muslims [8]. Since the primary reason for this centuries-old practice in Jewish and Muslim communities is religious tradition, it is unlikely to be swayed by scientific evidence or ethical discussions. Instead, it is important to focus on ensuring that circumcisions are performed as safely as possible in these communities.

Unlike Jewish traditions where circumcision is clearly advised on the seventh day after birth, Islamic traditions do not provide specific recommendations on the timing of the ritual; hence the age of circumcision among Muslims varies widely [1, 9]. The advantages of early circumcisions, preferably during the neonatal period, have been clearly established in literature. These include decreased incidence of urinary tract infections (UTI), faster healing, lower complications rates, and greater cost efficiency [2, 7, 8, 10]. A systematic review of prospective studies document complication rates ranging from 1.4 to 16% in Muslim-majority countries [8]. There appears to be a clear correlation between complications and increasing age [11–13]. Other factors contributing to complications include the training and experience of providers and the device used as well as the sterility of the setting where circumcisions are performed [8].

A better understanding of the perceptions and practices around male circumcision would help guide a strategy for designing and implementing safe circumcision programs in Muslim-majority settings, with the potential to benefit an annual birth cohort of 20–25 million boys. Since either one or both parents are the primary decision makers regarding circumcisions, this study aims to understand parental perceptions regarding circumcision and its timing and factors associated with delay in circumcision in Karachi, Pakistan.

## 2. Materials and Methods

### 2.1. Study Setting and Design

A cross-sectional study was conducted from May to November 2013 at the Indus Hospital in Karachi, Pakistan. The study population consisted of adult men and women (aged 50 years or below) who had at least one male child less than 18 years at the time of interview. The participants were identified while waiting in the paediatric outpatient area of the hospital.

### 2.2. Sample Size

Sample size was calculated on the basis of findings of a Pakistani cross-sectional survey that estimated 26% boys are circumcised as neonates [14]. Taking a desired precision of 0.15 with a 95% confidence interval, a sample size of 486 was required to cover the study objectives. To account for refusals, a final sample size of 500 was taken.

### 2.3. Data Collection

The study team approached eligible parents in the hospital's paediatric outpatient clinic and informed consent was obtained. In view of high illiteracy rates in our population, the Urdu version of the consent form was administered orally by the health workers to the respondents. Parents were interviewed using a precoded study questionnaire. The questionnaire was developed in English, translated into Urdu (the national language), and then backtranslated into English. It was pretested on 30 persons to ensure language comprehension. Trained research assistants used the Urdu questionnaire to conduct face-to-face interviews. No names or personal identifiers were recorded in order to ensure confidentiality.

During the interview, parents answered questions about demographics, education and income, and details of circumcision of male children in the household with a focus on real and perceived delays in their child's circumcisions and perceptions about appropriate age and reasons and benefits and complications of the procedure.

The study had approval from the hospital's institutional review board for human subject research.

### 2.4. Data Analysis

Actual delay in circumcision was calculated using age at interview for uncircumcised children and age at circumcision of those who were circumcised. Actual delay in circumcision was defined as children who were circumcised after two months or children who were not circumcised but had crossed the age of two months.

SPSS 21 was used for data analysis. For descriptive analysis, means and standard deviations were reported for continuous variables. For inferential analysis, chi-square, Fisher-exact, or likelihood ratio tests were applied to check for significant association between categorical variables, as appropriate. Delay in circumcision was the outcome of interest. Any association with a *P* value of 0.25 or less was included to build a parsimonious model using multiple logistic regression with backward stepwise elimination to test the significance at each step. In addition, biologically or socially significant variables were included in the multiple logistic regression analysis. *P* value < 0.05 was considered significant.

## 3. Results

Five hundred parents with at least one male child less than 18 years of age were interviewed regarding circumcision status of their male children. The median (IQR) age of parents was 31 (26–38) years (Table 1), with a median age of 36 years for fathers and 28 years for mothers. Mean household members were 7 (SD ±5.52), with a mean of 2 (SD ±1.1) male children per household. Participants were predominantly Muslims (92.8%), followed by Christians (7%) and Hindus (0.2%). Most of the participants (73.4%) came from the hospital's catchment area. Language spoken by the majority was Urdu (43.2%), followed by Punjabi (27.2%), Sindhi (11.8%), and Pushto (7.4%) (Table 1). Nearly one-quarter of the fathers (22.4%) and over one-third of the mothers (38.4%) were illiterate. Religious requirement was cited as the primary reason for circumcision of their child by 92.6% including 4 non-Muslims, medical reasons were cited by 5.8%, and a perception of improved sexual satisfaction was cited by 4%.

These 500 participants had a total of 987 male children below age of 18 years, but complete information was available for 954 children; therefore all further analysis was done on this group. Of these 954 children, 728 (76.3%) were reportedly circumcised at the time of the interview; the remaining 226 (23.7%) were not circumcised according to the parents at the time of interview. Of the 728 circumcised children, one-third of the children had delayed circumcision, that is, after two months of age. Of the 226 uncircumcised children, 7% were below two months of age and thus did not meet the criteria of delayed circumcision. The remaining 210 children (93%) had delay in circumcision. Thus, of all 954 children, half (54.1%) had delayed circumcision (Table 3). Age at circumcision ranged from 10 days to 13 years. Median (IQR) age at circumcision was 1 (0.27–12) month (Table 1). The median amount paid for a circumcision was PKR 300 (approximately USD 3) and ranged between PKR 0 and 3000 (USD 0–30). Only 4.3% circumcisions were performed free of cost (Table 1).

The most frequently performed method of circumcision was an open method using a blade with no suturing and no general anaesthesia (69.6%, mean circumcision age: 11.9 ± 22 months) followed by Plastibell circumcision (18.8%, mean circumcision age: 10.3 ± 25 months). In 0.3% of cases, formal open circumcisions were performed under general anaesthesia (Table 2).

Complications were reported in 88 circumcisions (12.1% of all circumcised children) with swelling (*n* = 42), mild bleeding (*n* = 21), significant bleeding (*n* = 9), inadequate circumcision (*n* = 7), infection (*n* = 1), and mortality (*n* = 1) being reported. The circumstances and correlation of the circumcision with death was not detailed. Complications were reported in 18.2% of the Plastibell group as compared to 10.1% in the open method group; this result was statistically significant (*P* = 0.041, Table 2).

Of the 728 circumcised boys, a delay in circumcision was seen in 49.1% (*n* = 313) of facility-delivered babies as compared to 60.4% (*n* = 173) of home-delivered babies (Table 3(b)). Nearly eighty percent of the boys were circumcised at a health facility (*n* = 577), and 20% were circumcised at home. There was a delay in circumcision in 38.5% of boys circumcised at a facility, as compared to a delay in 54.5% of those circumcised at home. The relationship between venue of birth and circumcision is detailed in Table 3(d), where it is seen that a majority of boys born at a health facility are circumcised at a health facility and only half of the boys born at home are circumcised at home.

When parents were asked about the ideal age for circumcision, 81.2% of parents were of the opinion that circumcisions should be done within 60 days of birth, that is, within our defined cut-off for a timely circumcision (Table 4). A third 30.2% of respondents found it acceptable to have their child circumcised by a female health provider. More fathers were accepting of female providers (33.6%) versus mothers (26.8%), but this was not found to be statistically significant (Table 4). In the majority that felt it was not acceptable for a female provider to perform circumcision, the reasons cited were that this was not a task to be done by women (43.4%), women providers were not experienced in this procedure (34.7%), they did not like a circumcision to be performed by a woman (20.1%), this was against religious dictates (0.7%), or they had never heard of a woman doing a circumcision (0.7%).

Almost three-quarters of the respondents were of the opinion that circumcisions should be performed by trained medical practitioners (either paediatric surgeons or paediatricians) (Table 4). When asked what in their opinion was a reasonable fee to charge for a circumcision, the response was evenly divided between free of cost (24.8%), less than PKR 100 (34.8%), and more than PKR 100 (33.6%). In the last category, the acceptable amount quoted ranged from PKR 100 to 2000 (conversion rate USD 1 = PKR 104; Table 4).

Most respondents (89.6%) felt there were medical benefits to circumcision (Table 4), with decreased UTI (42.6%), improved hygiene (27.8%), decreased STI (24.3%), reduced risk of cancer (4.7%), and being generally good for health (0.6%) being quoted.

A little over half of the respondents (56.6%) had heard about complications subsequent to a circumcision procedure. The complications reported included inadequate circumcision requiring a revision (18.3%), hematoma or swelling (17.5%), infection (11.9%), prolonged and significant pain (8.5%), mild (3.5%) and significant (8.0%) bleeding, deaths (1.5%), and delayed wound healing (0.3%).

To assess the risk factors associated with delay in circumcision, sixty-five households with a mixture of delayed circumcisions as well as no delay in circumcisions were excluded. Only households with no delays (*n* = 215) or all delays (*n* = 205) were included for the multiple logistic regression analysis. In the final MLR model, being non-Muslim (aOR: 5.36; 95% CI: 1.99–14.4); belonging to either a Sindhi (aOR: 2.65 95% CI: 0.87–8.08) or Balochi (aOR: 1.21 95% CI: 0.20–7.22) household; and a perception of circumcisions ideally being performed at an age older than 2 months (aOR: 3.02 95% CI: 1.60–5.72) were factors associated with delay in circumcision after adjusting for parent's education (Table 5). Being from a Pakhtoon household was protective against delayed circumcisions (aOR: 0.2 95% CI: 0.1–0.7).

## 4. Discussion

To our knowledge, this is the first study reviewing parental perceptions on circumcision in the Pakistani context. In a Muslim-majority country, the primary reason for opting to circumcise male offspring is religious requirement. In Islamic tradition, although male circumcision is recommended practice (*sunnah*), there is no clear directive on when this procedure is to be performed. Therefore, there is a wide variation in practice in Muslim communities [9, 15], with children often being circumcised in late childhood or early adolescence. Based on scientific recommendations, circumcisions are best performed during the newborn period when there is the least risk and the greatest medical benefit [16]. Complication rates increase with increasing age [8] even within the first year of life [8, 11, 13]. In high-income populations where circumcisions are routine, this procedure is performed in the neonatal period or early childhood, with complication rates generally reported to be less than 1% [3]. However, most countries with large Muslim populations are not geared to systematically providing circumcisions during the neonatal period as part of routine health care. As a consequence, the complication rates can be much higher, ranging from 0 to 16% [8, 14]. Interestingly, although the majority of our study population circumcised their children to meet a religious requirement, nearly all the respondents also believed that there were medical benefits to the procedure. Reinforcing this perception with education and awareness about the benefits of early circumcision could be a good strategy in promoting timely circumcisions in Muslim populations. The approach would need to be adapted to address cultural and traditional norms that are prevalent in different communities.

Though a religious recommendation, our study indicates that cultural and social norms play an important role in the timing of circumcision. Within Pakistan, there are certain ethnic populations, like the Balochis and Sindhis, that regard circumcision as a rite of passage or a cause for celebration, often saving for months or years before they can afford to host a lavish party to celebrate their son's circumcision. Our study results show that belonging to these ethnic groups is a risk factor for delayed circumcision. In contrast, it appears to be customary for Pakhtoon families to circumcise their babies early, often within the first week of life. Understanding the complex and varying traditions that may exist within one country or region is important in drafting culturally relevant and locally acceptable educational material and awareness campaigns.

In our study, majority of the boys whether born in a hospital or at home were circumcised at a health facility although almost half of these circumcisions were delayed. Recent national data suggests that this is a reflection of the overall situation in Pakistan, with 67.9% of urban and 40.1% of rural deliveries taking place in facilities and a strong correlation seen between income level and facility deliveries [17]. By providing information to parents during antenatal and early postnatal period, health facilities can play an important role in promoting early and safe circumcisions. Parent education and awareness through facility-based programs as well as through the existing network of lady health workers, vaccinators, and general practitioners, particularly in rural settings, could help promote early circumcisions. A community based Plastibell circumcision service for the Muslim population in UK appeared to be successful in meeting local needs [18]. Despite a reported complication rate of 5.5%, parental satisfaction continued to be very high.

Circumcisions done in the newborn and early infancy period are simple to perform in an outpatient setting, and a trained health worker can perform this procedure safely [2, 18–20]. Moreover, this is the least costly option [3]. Jayanthi et al. estimated that a circumcision performed under general anaesthesia in an operating room setting costs $1,805 as compared to $196 for early infant circumcisions performed with local anaesthesia in an office setting [10]. This has important implications for Pakistan, with an annual male birth cohort of 2.3 million boys [21], an estimated 95–98% of whom are Muslim and will undergo circumcision at some point during their childhood or adolescence. Most families (94%) in our setting end up paying out of pocket for this procedure. When asked, 60% of parents would prefer that this procedure be done either free of cost or for a nominal fee of less than PKR 100 (approximately USD 1). In a resource constrained setting like ours, it is advantageous to promote early circumcisions that can be performed safely and at lower costs to an already overburdened health system. Parents in our study preferred to have this procedure performed by trained medical providers. Given the acute shortage of medical and nursing personnel in the country, a practical and acceptable alternative may be to train health workers to perform circumcisions safely. These health workers may either be paramedical staff with short-term training or experience in working in health facilities or community health workers. Although our study suggests that parents are generally not accepting of women providers performing the procedure, the corresponding author of this study is a female and has been performing circumcisions for the past 25 years without a single incident where the family refused or was unhappy based on the author's gender. It would appear that good training and expertise would take precedence over the gender of the provider.

There are several methods that can be used to circumcise a child. Familiarity and acceptance by both provider and parents are important determinants when deciding on the most appropriate method to use in a population-based program. The Plastibell method is one of the four techniques recommended by the WHO for childhood circumcisions [22]. It has been shown to be a safe technique in different settings [23–25], with comparable results to other methods. It is the method generally used by paediatric surgeons, paediatricians, and general surgeons across Pakistan to circumcise infants and is the technique that is taught to residents and trainees in accreditation programs and teaching hospitals [18, 26, 27]. Open method circumcisions, like the freehand technique, are performed by specialists in older boys in hospital settings with either local or general anaesthesia. The real challenge is that a majority of circumcisions in Pakistan are performed by untrained and unqualified persons using methods like the bone cutter technique or simply slicing off the foreskin with no proper hemostasis in unsterile conditions. Plastibell circumcisions are also being performed by people who have no formal training. Understandably, it is these substandard techniques by untrained providers that lead to the high complication rates seen in our setting. In our study there was a reported complication rate of 12%, with a higher complication rate seen in Plastibell circumcisions. In a prospective hospital based study from Pakistan, Rehman et al. [28] reported adverse events in 16% of circumcisions done using the free hand or bone cutter techniques. In contrast, in two retrospective reviews from hospitals in Pakistan, Iftikhar and Rafiq reported 0.6% and 2% complication rates, respectively, using the Plastibell technique [26, 29]. The importance of thorough training, meticulous technique, and careful monitoring is critical in ensuring satisfactory outcomes. Awareness campaigns informing the population about the benefits of early infant circumcision using a safe and sterile method could help change traditional practices.

Over half the respondents had heard of complications occurring subsequent to circumcisions. Inadequate removal of foreskin was commonly reported, with the child being put through a revision at a later stage. Residual foreskin leaves the individual susceptible to HIV infection [30], and hence one of the major epidemiological advantages of male circumcision may be compromised. Other minor and major complications are also frequently encountered, making it all the more important to train providers to use correct and standardized techniques.

One of the study limitations in this type of survey can be a recall bias; especially in those parents with multiple boys or older boys who may not remember the details of the procedure. Furthermore, there is a possibility that, in a society where circumcision is a religious obligation, some parents may not have provided an honest response.

## 5. Conclusion

In a country where circumcision is a routine practice, the important aspect is to focus on ensuring that circumcisions are performed as safely as possible and as early as possible. Birth venue can play a vital part in ensuring timely circumcision. Since a large proportion of circumcision delays take place in boys delivered at home, involvement of traditional birth attendants or trained community health workers to perform safe and early circumcision should be considered. Health facilities should also consider offering circumcision as part of the delivery package to ensure timely circumcision.

## Figures and Tables

**Table 1:** Demographic characteristics of participants, *n* = 500.

Age in years; median (IQR)	
Father	36 (30–40.2)
Mother	28 (25–32)
Overall	31 (26–38)
Ethnicity; *n* (%)	
Urdu speaking	216 (43.2)
Punjabi/Saraiki/Hazara	136 (27.2)
Sindhi	59 (11.8)
Pakhtoon	37 (7.4)
Balochi	11 (2.2)
Others	41 (8.2)
Formal years of education; mean (SD)	
Fathers' education	6.6 (4.7)
Mothers' education	5.3 (4.9)
Circumcision status; *n* (%)	
Circumcised	728 (76.3)
Uncircumcised	226 (23.7)
Delay, overall	516 (54.1)
(i) Among circumcised subjects	306 (42.0)
(ii) Among uncircumcised subjects	210 (92.9)
Circumcision age, overall, months	
Median (IQR)	1 (0.27–12)
Min–Max	0.03–156
Circumcision age – among those with delay	
Median (IQR)	12 (7–36)
Min–Max	2.5–156
Circumcision age, among those with no delay	
Median (IQR)	0.42 (0.23–1)
Min–Max	0.03–2
Age parent's perceived a delay had occurred	
Months, median (IQR)	7 (6–24)
Cost of circumcising a child, PKR^§^	
Median (IQR)	300 (200–500)
Min–Max	0–3000

^§^Conversion USD 1 = PKR 104 (July 2016).

**Table 2:** Type and age at circumcision with complication rates.

Type of circumcision	Age at circumcision				Overall complications^*∗*‡^
	≤2 months	>2 months to 1 year	>1 year	Total	
Plastibell (18.8%)	91 (66.4)	25 (18.2)	21 (15.3)	137	25 (18.2)
Blade/open method without GA (69.6%)	282 (55.6)	115 (22.7)	110 (21.7)	507	51 (10.1)
Open method with GA (0.3%)	0 (0.0)	2 (100)	0 (0.0)	2	1 (50.5)
Do not recall type (11.3%)	49 (59.8)	20 (24.4)	13 (15.9)	82	11 (13.4)
*Total*	*422 (58.0)*	*162 (22.3)*	*144 (19.8)*	*728*	*88 (12.1)*

^*∗*^ *P* value (0.041) < 0.05. ^‡^Likelihood ratio chi-square test.

**(a):** 

Circumcision status^*∗∗*†^	Delay status—all boys		
	No delay, *n* (%)	Delay *n* (%)	Total
Circumcised	422 (58.0)	306 (42.0)	728 (100)
Uncircumcised	16 (7.1)	210 (92.9)	226 (100)
Total	438 (45.9)	516 (54.1)	954

**(b):** 

Birth venue^*∗∗*†^	Delay status—all boys		
	No delay *n* (%)	Delay *n* (%)	Total
Health facility	325 (50.9)	313 (49.1)	638 (100)
Home	113 (39.5)	173 (60.5)	286 (100)
Missing info	0 (0)	30 (100)	30 (100)
Total	438 (45.9)	516 (54.1)	954 (100)

**(c):** 

Circumcision venue^*∗∗*†^	Delay status—among the circumcised subjects		
	No delay *n* (%)	Delay *n* (%)	Total
Health facility	355 (61.5)	222 (38.5)	577 (100)
Home	66 (45.5)	79 (54.5)	145 (100)
Do not know/missing	1 (16.7)	5 (83.3)	6 (100)
Total	438 (45.6)	516 (54.1)	728 (100)

**(d):** 

Venue of birth^*∗∗*†^	Circumcision venue		
	Health facility (*n* = 577)	Home (*n* = 145)	Missing (*n* = 6)
Health facility	454 (89.2)	51 (10.0)	4 (0.8)
Home	123 (56.2)	94 (42.9)	2 (0.9)

^*∗∗*^ *P* value < 0.0001. ^†^Chi-square test.

**Table 4:** Perception of parents.

	Relationship to child		
	Father *n* = 250	Mother *n* = 250	Total *n* = 500
Parental knowledge of ideal circumcision age^*∗*†^			
Ideal age ≤60 days	193 (77.2)	213 (85.2)	406 (81.2)
Ideal age >60 days	53 (21.2)	31 (12.4)	84 (16.8)
Do not know/missing	4 (1.6)	6 (2.4)	10 (2)
Acceptance of female performing circumcision^†^			
Not acceptable	166 (66.4)	183 (73.2)	349 (69.8)
Acceptable	84 (33.6)	67 (26.8)	151 (30.2)
Opinion on who should perform circumcision?^*∗*†^			
Paediatric surgeon/paediatrician	170 (68)	200 (80)	370 (74)
Others	68 (27.2)	41 (16.4)	109 (21.8)
Do not know/missing	12 (4.8)	9 (3.6)	21 (4.2)
Opinion that circumcision has medical benefits^*∗*†^			
Yes, has medical benefits	215 (86)	233 (93.2)	448 (89.6)
No medical benefits	17 (6.8)	4 (1.6)	21 (4.2)
Do not know/missing	18 (7.2)	13 (5.2)	31 (6.2)
Reasonable amount for circumcision^*∗∗*†^			
Free	48 (19.2)	76 (30.4)	124 (24.8)
1–100	80 (32)	94 (37.6)	174 (34.8)
101–2000	110 (44)	58 (23.2)	168 (33.6)
Do not know/missing	12 (4.8)	22 (8.8)	34 (6.8)

^*∗*^ *P* value < 0.05. ^*∗∗*^*P* value < 0.0001. ^†^Chi-square test.

**Table 5:** Risk factors associated with delay in circumcision.

	Delay in all children^a^	
	Unadjusted OR (CI)^b^	Adjusted OR (CI)^c^
Religion		
Non-Muslim	4.1(1.7–9.7)^*∗*^	5.4(2.0–14.4)^*∗*^
Muslim	Ref	Ref
Races		
Urdu speaking	0.4(0.2–0.9)^*∗*^	0.6 (0.2–1.3)
Punjabi/Hazara/Siraiki	0.6 (0.3–1.3)	0.4 (0.2–1.0)
Pakhtoon	0.2(0.1–0.7)^*∗*^	0.2(0.1–0.7)^*∗*^
Sindhi	3.4(1.2–9.5)^*∗*^	2.7 (0.9–8.1)
Balochi	2.2 (0.4–12.1)	1.2 (0.2–7.2)
Others	Ref	Ref
Parents years of formal education		
Father	0.94(0.9–0.98)^*∗*^	0.96 (0.91–1.01)
Mother	0.92(0.88–0.96)^*∗∗*^	0.93(0.88–0.98)^*∗*^
Total number of male children	1.15 (0.96–1.38)	—
Venue of birth of last child		—
Clinic	0.99 (0.33–3.02)	
Home	1.48 (0.95–2.31)	
Hospital	Ref	
Parents opinion of ideal age of circumcision		
Ideal age >60 days	4.67 (2.62–8.33)	3.02(1.60–5.72)^*∗*^
Ideal age ≤60 days	Ref	Ref

^a^Reference category: no delay in all children. ^b^Univariate binary logistic regression. ^c^Multivariate binary logistic regression. ^*∗*^*P* < 0.05; ^*∗∗*^*P* < 0.001.
